# Photobiomodulation as a Hypothetical Strategy to Reverse Botulinum Toxin Effects: Exploring the Neuroregenerative Mechanisms and Translational Potential

**DOI:** 10.3390/life15081206

**Published:** 2025-07-28

**Authors:** Rodrigo Álvaro Brandão Lopes-Martins, Francisco Gonzalez-Lima, Sérgio Gomes da Silva, Patrícia Sardinha Leonardo, Cristiane Soncino, Roberto Fernandes Pacheco, Carolina Lúcia de Oliveira e Oliveira, Fabrizio dos Santos Cardoso

**Affiliations:** 1 Hospital do Câncer de Muriaé, Fundação Cristiano Varella (FCV), Muriaé 36888-233, MG, Brazil; sergio.gomes@fcv.org.br (S.G.d.S.); fabrizio.cardoso@uniredentor.edu.br (F.d.S.C.); 2 Research and Innovation Department Redentor Afya University Center (UniREDENTOR Afya), Itaperuna 28300-000, RJ, Brazil; patricia.sardinha@uniredentor.edu.br; 3 Programa de Pós-Graduação em Bioengenharia, Universidade Brasil, São Paulo. Av. Carolina Fonseca 236, Itaquera – São Paulo 08230-030, SP, Brazil; 4 Texas Consortium in Behavioral Neuroscience, Departments of Psychology, Pharmacology and Toxicology, University of Texas at Austin, Austin, TX 78712, USA; gonzalezlima@utexas.edu; 5 Research Department, Hospital São Vicente de Paulo (HSVP), Bom Jesus do Itabapoana 36010-570, RJ, Brazil; 6 Laboratory of Neurosciences, Centro Universitário FAMINAS, Muriaé 36880-000, MG, Brazil; 7 Research Department, Faculdade de Tecnologia em Saúde (FATESA), Ribeirão Preto 14026-583, SP, Brazil; cris.soncino@fatesa.edu.br; 8 Instituto Dr. Ellev, Rua Conde de Linhares 112 Bairro, Cidade Jardim, Belo Horizonte 30840-030, MG, Brazil

**Keywords:** botulinum toxin, photobiomodulation, neuromuscular recovery, synaptic regeneration, aesthetic complications, neuroplasticity

## Abstract

**Background:** Botulinum toxin type A (BoNT/A) is widely used in both clinical and aesthetic settings to induce temporary neuromuscular paralysis by inhibiting acetylcholine release. Although generally regarded as safe and effective, complications such as iatrogenic ptosis or facial asymmetry may occur and persist for several weeks or even months, with no standardized method currently available to accelerate recovery. **Objective:** This article explores the hypothesis that photobiomodulation (PBM)—a non-invasive modality recognized for its neuroregenerative potential—may facilitate the reversal of BoNT/A-induced neuromuscular blockade. **Discussion:** PBM enhances mitochondrial activity by stimulating cytochrome c oxidase in nerve and muscle tissues, thereby increasing ATP production and modulating intracellular signaling pathways associated with neuroplasticity, cell survival, and synaptogenesis. Preclinical studies have demonstrated that PBM can upregulate neurotrophic factors (e.g., BDNF, NGF), enhance SNAP-25 expression, and promote structural remodeling of neurons in both young and aged brains. These mechanisms are biologically consistent with the regenerative processes required for recovery from BoNT/A-induced effects. While controlled clinical trials for this specific application are currently lacking, anecdotal clinical reports suggest that PBM may accelerate functional recovery in cases of BoNT/A-related complications. **Conclusions:** Although this approach has not yet been tested in clinical trials, we propose that photobiomodulation may hypothetically serve as a supportive strategy to promote neuromuscular recovery in patients experiencing adverse effects from BoNT/A. This hypothesis is grounded in robust preclinical evidence but requires validation through translational and clinical research.

## 1. Introduction

Botulinum toxin type A (BoNT/A) is widely used in clinical practice for both therapeutic and aesthetic purposes [1,2,3]. Its primary mechanism of action occurs at the neuromuscular junction, where it inhibits the release of acetylcholine—a neurotransmitter essential for muscle contraction. This inhibition results in temporary muscular paralysis, which is gradually reversed as nerve terminals regenerate and synaptic connections are reestablished [4,5,6]. Although BoNT/A is generally considered safe, complications such as eyelid ptosis, facial asymmetry, and unintended diffusion of the toxin occur in up to 5–6.5% of aesthetic applications, particularly in the upper face [7]. These effects, while temporary, can persist for several weeks and significantly impair visual function, facial appearance, and patient satisfaction [7,8]. In therapeutic contexts, adverse events such as dysphagia, excessive muscle weakness, or suboptimal outcomes due to toxin spread have also been reported [9]. Despite their reversibility, no standardized intervention currently exists to actively accelerate recovery from these events, and their impact on function and quality of life should not be underestimated.

Photobiomodulation (PBM), also referred to as low-level laser therapy [10], has emerged as a promising modality in regenerative medicine [11,12,13]. Studies have shown that PBM stimulates mitochondrial cytochrome c oxidase (CCO), resulting in increased adenosine triphosphate (ATP) production in nerve and muscle tissues. This bioenergetic enhancement supports nerve regeneration, promotes vascularization, and facilitates neuroplasticity, including the formation of new synapses [11,14,15,16]. These effects are attributed to the activation of intracellular signaling pathways that promote neuronal survival and growth [17,18].

Based on these mechanisms, it is plausible that PBM may accelerate the reversal of botulinum toxin-induced effects, thereby facilitating earlier recovery of neuromuscular function. This hypothesis is particularly relevant in aesthetic contexts—such as cases of iatrogenic eyelid ptosis—as well as in clinical situations where early termination of the toxin’s effect is desirable. In this context, the present article aims to examine the feasibility of this approach by exploring the cellular and molecular mechanisms potentially involved in PBM-induced synaptic regeneration and its possible clinical applications.

### 1.1. Mechanism of Action of BoNT/A

Botulinum toxin type A (BoNT/A), produced by Clostridium botulinum, exerts its clinical effect by inducing temporary neuromuscular paralysis through the inhibition of acetylcholine release at the neuromuscular junction. This highly specific action occurs via the following multistep process: binding to presynaptic cholinergic terminals, internalization via endocytosis, translocation into the cytosol, and cleavage of key proteins in the SNARE complex responsible for synaptic vesicle fusion [19,20]. Specifically, BoNT/A cleaves the synaptosomal-associated protein of 25 kDa (SNAP-25), thereby preventing the exocytosis of acetylcholine-containing vesicles and ultimately leading to functional muscle denervation [3,19].

Recovery from BoNT/A-induced blockade is a slow biological process involving axonal sprouting, synaptic remodeling, and the re-establishment of neuromuscular transmission [21,22]. The duration of BoNT/A’s clinical effect—typically ranging from several weeks to a few months—varies depending on the dose, injection site, and individual tissue response. While this pharmacological profile is desirable for many clinical indications, prolonged adverse effects such as iatrogenic ptosis or facial asymmetry can persist beyond the expected therapeutic window and currently lack established strategies for resolution.

In this context, photobiomodulation (PBM) emerges as a biologically plausible approach to support the recovery process. PBM has been shown to promote synaptic repair and upregulate proteins directly involved in synaptic function, including SNAP-25 [11,23]. Additionally, PBM enhances mitochondrial function and stimulates the expression of neurotrophic factors that facilitate axonal regeneration and synaptogenesis [17,18,24,25]. These cellular effects directly align with the regenerative mechanisms required to reverse BoNT/A-induced neuromuscular silencing, suggesting that PBM may accelerate the intrinsic recovery process. Establishing this mechanistic convergence provides the biological foundation for our hypothesis that PBM could be investigated as a supportive strategy to reduce the duration or severity of BoNT/A-related complications.

### 1.2. Mechanisms of Photobiomodulation in Neuromuscular Regeneration

Photobiomodulation (PBM) involves the use of red to near-infrared light to stimulate mitochondrial function and trigger regenerative signaling cascades in neural and muscular tissues. Its primary target is cytochrome c oxidase (CCO), a key enzyme in the mitochondrial respiratory chain, whose activation increases ATP synthesis and modulates redox-sensitive signaling pathways such as PI3K/Akt and MAPK/ERK [15,17,26,27,28]. These effects promote neuronal survival, axonal sprouting, and synaptic plasticity, particularly in models of trauma and neurodegeneration [18,24,25].

Several preclinical studies have shown that PBM can enhance the expression of neurotrophic factors (e.g., BDNF, NGF), stimulate synaptogenesis, and improve metabolic efficiency in both young and aged brains [16,29,30]. In muscle tissue, PBM has been associated with increased resistance to fatigue, improved performance, and protection against chemically induced myonecrosis [31,32,33,34,35,36,37]. These findings have contributed to the hypothesis that PBM could support recovery from botulinum toxin type A (BoNT/A)-induced neuromuscular blockade.

However, it is important to clearly distinguish between models of injury or degeneration and the pharmacological mechanism of BoNT/A. The toxin exerts its effect by cleaving SNAP-25—a vesicle fusion protein essential for acetylcholine release—through an intracellular, zinc-dependent protease mechanism [19,20]. This cleavage is highly specific and irreversible. To date, no evidence demonstrates that PBM can neutralize BoNT/A activity, degrade the toxin, or restore SNAP-25 once cleaved. Although some studies report increased SNAP-25 expression following PBM exposure [11], these were conducted in models of denervation or trauma, not in the context of BoNT/A-induced enzymatic inhibition.

Therefore, the potential benefit of PBM in this context is not as a direct antagonist to the toxin, but rather as a hypothetical adjunct capable of accelerating regenerative processes once BoNT/A begins to lose its intracellular efficacy. In other words, PBM may support the natural course of recovery by enhancing synaptic reorganization, axonal sprouting, and neuroenergetic efficiency after the toxin’s primary activity has subsided.

It is also important to acknowledge the broader limitations of PBM in translational applications. First, PBM exhibits a biphasic dose–response curve: insufficient dosages may be ineffective, while excessive exposure may paradoxically inhibit cellular activity [27]. Second, the literature reveals inconsistent findings, with several human trials and meta-analyses reporting null or inconclusive results [38], raising concerns about reproducibility, particularly in clinical settings. Third, the safety profile of PBM in aesthetic facial regions—especially near the eyes—remains unestablished. Given that BoNT/A is often administered in periorbital muscles, careful consideration of dosage, wavelength, and target depth is essential.

Finally, the intracellular pharmacokinetics of BoNT/A are governed by well-characterized processes—including endosomal internalization, SNARE protein cleavage, and vesicular component turnover—which typically require 3 to 4 months for full recovery [22]. These processes are not modifiable by mitochondrial stimulation alone. As such, whilst PBM may support the structural and metabolic conditions favorable to regeneration, it cannot override the intrinsic biological timeline of the toxin.

In summary, the rationale for exploring PBM in BoNT/A-related complications should be framed within its supportive—not antagonistic—potential. Rigorous experimental models that replicate the enzymatic blockade induced by BoNT/A are essential to validate this hypothesis and inform future translational studies.

## 2. Results

A growing body of preclinical and translational research supports the hypothesis that photobiomodulation (PBM) exerts significant neuromodulatory and neuroregenerative effects relevant to the reversal of BoNT/A-induced neuromuscular blockade. Table 1 summarizes key studies that provide experimental evidence supporting the biological mechanisms and clinical plausibility of this approach.

These studies, encompassing both animal and human models, demonstrate that PBM can enhance mitochondrial function, upregulate cytochrome c oxidase (CCO) activity, increase the expression of neurotrophic factors such as BDNF and NGF, and promote synaptogenesis and axonal regeneration. Additionally, PBM has been shown to improve functional outcomes in models of neural trauma, neurodegenerative diseases, and muscle injury. Notably, some studies—including those from independent research groups—have reported upregulation of SNAP-25, BoNT/A’s molecular target, following PBM exposure, reinforcing its potential to modulate toxin-induced synaptic silencing [11,23,25].

Taken together, the evidence suggests that PBM induces a constellation of cellular and molecular changes that are biologically consistent with the processes required for synaptic recovery, including dendritic remodeling, neurovascular support, and resolution of inflammation. These effects have been observed across various experimental conditions and tissue types, underscoring the robustness of PBM’s mechanisms and its potential utility in mitigating adverse outcomes associated with BoNT/A treatments.

## 3. Discussion

BoNT/A exerts its pharmacological effect by cleaving SNAP-25, a synaptic vesicle fusion protein essential for acetylcholine release. This enzymatic activity, mediated by a zinc-dependent protease, results in flaccid paralysis. Functional recovery typically requires 3 to 4 months and depends on axonal sprouting and synaptic remodeling, rather than reversal of the enzymatic cleavage [19,22].

PBM has demonstrated the capacity to enhance mitochondrial function, increase neurotrophic signaling, and stimulate regenerative pathways in models of nerve injury, muscle fatigue, and neurodegeneration [12,15,16,17,24,25,27]. These biological effects include activation of cytochrome c oxidase, increased ATP production, and modulation of intracellular pathways such as PI3K/Akt and MAPK/ERK [27]. Preclinical studies also report upregulation of BDNF, enhanced synaptogenesis, and improved metabolic function in injured tissues [29,33]. However, the applicability of these findings to the specific pharmacodynamics of BoNT/A remains unproven.

Based on these biological foundations, we hypothesize that PBM could facilitate earlier resolution of BoNT/A-induced neuromuscular blockade by accelerating the intrinsic regenerative processes required for synaptic reestablishment. Specifically, the upregulation of SNAP-25 and enhanced neurotrophic support may help counteract the synaptic silencing induced by BoNT/A, while improved local vascularization and reduced inflammation may further contribute to a favorable recovery environment. Although this hypothesis has not yet been validated in controlled experimental settings, anecdotal clinical reports suggest that PBM may shorten the duration of adverse effects related to botulinum toxin, such as iatrogenic ptosis or facial asymmetry. Given its favorable safety profile and mechanistic plausibility, PBM warrants systematic investigation as a potential therapeutic adjunct for managing BoNT/A-related complications. Nevertheless, any intervention aimed at accelerating recovery from botulinum toxin type A (BoNT/A) must be considered with caution. In therapeutic contexts such as cervical dystonia, spasticity, or chronic migraine, a reduction in the duration of BoNT/A’s effects could compromise symptom control and overall treatment efficacy [39]. For instance, dysphagia, muscle weakness, and suboptimal symptom relief have been reported as adverse effects even under standard dosing conditions [40]. Therefore, the potential application of photobiomodulation should be restricted to cases involving adverse or undesired outcomes, particularly in aesthetic practice where complications like eyelid ptosis and facial asymmetry can cause significant distress [41].

### Limitations and Future Directions

Several critical limitations must be acknowledged when considering PBM as a potential strategy for managing BoNT/A-associated complications. First, the molecular mechanism of BoNT/A is unique among neurotoxins as it targets SNAP-25 through intracellular cleavage. This effect is irreversible, and recovery depends on the natural turnover and regeneration of synaptic proteins rather than on the modulation of acute inflammatory or metabolic states [19,20]. Current PBM literature does not include models that directly replicate the pharmacodynamics of BoNT/A [17].

Second, PBM exhibits a biphasic dose–response relationship, with potential for subtherapeutic or even inhibitory outcomes depending on parameters such as wavelength, power density, and treatment frequency [27]. These parameters have not yet been optimized for use in patients recovering from BoNT/A-related complications, particularly in sensitive facial regions where aesthetic applications are most common. To date, no preclinical or clinical studies have defined specific PBM protocols for this indication, preventing the development of a scientifically grounded therapeutic approach. This gap underscores the importance of initial dose-finding and mechanistic studies prior to clinical implementation.

Third, although PBM has demonstrated promise in various clinical contexts, inconsistencies persist. A study by Stausholm et al. [38] highlighted substantial heterogeneity among PBM trials, with some reporting null or contradictory outcomes. The absence of randomized controlled trials evaluating PBM specifically for BoNT/A recovery further emphasizes the need for cautious interpretation.

Fourth, safety concerns related to PBM application near the eyes or cranial nerves must be carefully addressed. The periorbital region is anatomically delicate, and improper PBM use could result in unintended tissue effects or photothermal injury. Regulatory guidelines and dedicated clinical trials will be necessary to ensure safe application in aesthetic medicine.

In light of these considerations, we propose that PBM should be investigated as a supportive, time-dependent adjunct—not as a primary treatment—for select patients experiencing prolonged or undesirable effects from BoNT/A. Future research should include BoNT/A-specific experimental models, precise dosimetry studies, and well-designed clinical trials to assess not only efficacy but also safety and optimal treatment parameters.

**Figure 1:**
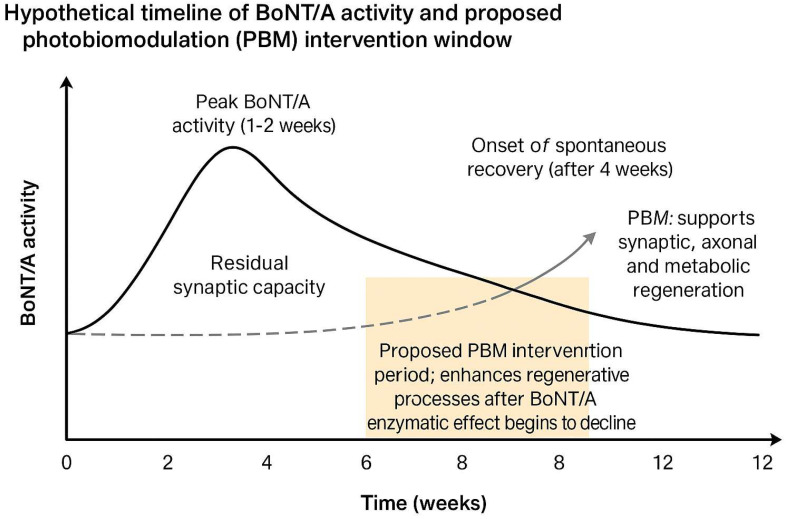
Hypothetical timeline of Botulinum Neurotoxin Type A (BoNT/A) activity and proposed photobiomodulation (PBM) intervention window. BoNT/A activity typically peaks within 1–2 weeks after injection and gradually declines over 3–4 months. The proposed PBM application window, between weeks 4 and 8, aims to enhance synaptic, axonal, and metabolic regeneration during the early recovery phase when BoNT/A enzymatic effects have diminished but neuromuscular reinnervation is still ongoing.

A schematic representation of the hypothetical time-aligned role of PBM in BoNT/A recovery is provided in Figure 1, illustrating the proposed therapeutic window during which PBM could enhance regenerative mechanisms after the enzymatic activity of BoNT/A begins to decline.

## 4. Conclusions

This hypothesis paper proposes that photobiomodulation (PBM), based on its well-established cellular and neuroregenerative mechanisms, may hypothetically serve as a supportive strategy to enhance recovery from adverse effects associated with botulinum toxin type A (BoNT/A) [15,17,24]. However, this proposal remains entirely theoretical, as no preclinical or clinical studies to date have demonstrated that PBM can accelerate the degradation of BoNT/A or reverse its enzymatic action on SNAP-25 [19,20].

We emphasize that this is a mechanistically grounded but untested hypothesis, and no clinical applicability should be inferred at this stage. Future research should begin with controlled preclinical studies employing rodent models of BoNT/A-induced focal paralysis to evaluate the potential effects of PBM on neuromuscular recovery. Relevant outcome measures may include electromyographic activity, expression levels of SNAP-25 and neuroplasticity-related markers such as BDNF and GAP-43, as well as functional motor assessments [29,33].

If supported by preclinical evidence, small-scale, double-blind, sham-controlled clinical trials could be considered in patients with prolonged iatrogenic complications such as ptosis or facial asymmetry. These trials must carefully define safety parameters, treatment dosimetry, and objective clinical endpoints [38].

In light of the above, we propose a translational roadmap starting with BoNT/A-specific animal models and time-course PBM protocols to identify optimal intervention windows. Once basic safety and efficacy parameters are established, early-phase clinical trials can investigate PBM as a potential therapeutic adjunct in real-world scenarios of toxin-related complications.

In conclusion, PBM presents a biologically plausible, non-invasive strategy that could hypothetically support synaptic regeneration and neuromuscular recovery in patients affected by undesired effects of BoNT/A. However, it must be rigorously validated through translational and clinical studies before it can be considered a viable medical intervention. Until such data are available, PBM should not be promoted as an evidence-based approach for BoNT/A-related complications. We hope this hypothesis stimulates further scientific exploration within the fields of aesthetic medicine and neuromodulation.

## Figures and Tables

**Table 1:** Key studies supporting the PBM hypothesis.

Author	Year	Experimental Model	Main Findings
[16]	2022	Rat cortical neurons	PBM altered neuronal morphology in the cerebral cortex, increasing dendritic branching and complexity.
[28]	2022	Young and aged rat brains	Chronic PBM increased CCO expression in young and aged brains, enhancing metabolic and neuroprotective activity.
[18]	2022	Aged rats	PBM improved spatial memory and reduced neuroinflammation in aged rats, suggesting cognitive and immunomodulatory benefits.
[17]	2021	Aged rat model	Improved intracellular signaling related to cell survival, memory, and glucose metabolism in aged brain after transcranial PBM.
[30]	2016	Human forearm (in vivo)	PBM increased CCO and oxygenated hemoglobin concentrations in muscle.
[25]	2015	Mouse traumatic brain injury model	PBM increased BDNF expression and synaptogenesis in a traumatic brain injury model in mice.
[23]	2014	Rat sciatic nerve injury model	Low-level laser irradiation enhanced functional recovery and nerve regeneration in a sciatic nerve crush model in rats.
[29]	2013	APP/PS1 Alzheimer’s mouse model	PBM reversed dendritic atrophy and increased BDNF in Alzheimer’s disease model, improving neuronal integrity.
[14]	2010	Rat superficial temporalis muscle of the face	PBM caused a dose-dependent increase in histochemical CCO activity in muscle fibers.
[35]	2009	Rat nerve-muscle model	PBM can effectively mitigate venom-induced myonecrosis and neuromuscular blockade.
